# The relaxant effect of the extract of *Crocus sativus* petal on Wistar rats airway smooth muscle and its possible mechanisms

**DOI:** 10.22038/ajp.2024.25150

**Published:** 2025

**Authors:** Sepide Behrouz, Arghavan Memarzia, Mohammad Hossein Eshaghi Ghalibaf, Amir Hossein Yazdi, Mohammad Hossein Boskabady

**Affiliations:** 1 Applied Biomedical Research Center, Mashhad University of Medical Sciences, Mashhad, Iran; 2 Department of Physiology, Faculty of Medicine, Mashhad University of Medical Sciences, Mashhad, Iran; 3 Department of Anatomy and Cell Biology, Faculty of Medicine, Mashhad University of Medical Sciences, Mashhad, Iran

**Keywords:** Relaxant effects, Crocus sativus petal, Airway smooth muscle histamine receptors, Calcium channels, Potassium channels

## Abstract

**Objective:**

Obstructive pulmonary diseases are characterized by airflow limitation secondary to airway wall thickening, airway narrowing and increased mucus secretion. Saffron (*Crocus sativus *L.) has shown different effects including anti-inflammatory, antioxidant, and immunomodulatory properties and promising effects for treating multiple disorders. In this study, the contribution of calcium and potassium channels, muscarinic and histamine (H_1_) receptors in the relaxant effect of the *C. sativus* petal extract on tracheal smooth muscle (TSM) was assessed.

**Materials and Methods:**

Fifty-four male Wistar rats divided in 8 groups, were studied. TSM was contracted by 10 μM methacholine or 60 mM KCl for 5 min, and the relaxant effects of cumulative concentrations of *C. sativus* petal extract (0.1, 0.2, 0.4 and 0.8 mg/ml), theophylline (0.2, 0.4, 0.6 and 0.8 mM) or 1 mL normal saline were tested. In non-incubated TSM and in TSM groups incubated with diltiazem, chlorpheniramine, propranolol, glibenclamide, atropine and indomethacin, the relaxant effects of the extract were evaluated.

**Results:**

The concentration-dependent relaxant effects of *C. sativus* petal extract on non-incubated TSM contracted by methacholine or KCl, were observed (for all, p<0.001). The relaxant effects of *C. sativus* petal extract in TSM incubated with chlorpheniramine and indomethacin, were significantly reduced compared to non-incubated tissues (p<0.05 to p<0.001).

**Conclusion:**

The results showed an obvious relaxation effect of the petal of *C. sativus* extract on TSM and suggest that inhibition of cyclooxygenase pathway and histamine receptors contribute to the extract relaxant effect of the extract.

## Introduction

Obstructive pulmonary diseases such as asthma are characterized by airflow limitation secondary to airway wall thickening, airway narrowing and increased mucus secretion (Agarwal et al., 2023). Asthma is a significant worldwide health issue that impacts about 250 million individuals and ranks second in causing death among chronic respiratory diseases and its prevalence has risen in the last ten years (Vos et al., 2020; D’Amato et al., 2016). Airway smooth muscle cells (ASMC) play a special role in the pathogenesis of obstructive pulmonary diseases. For example, exaggerated response of airways to non-specific stimuli or airways hyper-responsiveness (AHR) is closely linked to bronchial smooth muscle activity (Hassoun et al., 2022). *Ex-vivo* studies demonstrated a stronger contraction response of ASMC to histamine in asthmatic subjects compared to controls (Yamauchi and Ogasawara, 2019). Also, in asthmatic patients, the functional indices of the ASMC such as maximum capacity and shortening velocity increase significantly compared to healthy subjects (Sheng et al., 2022). Therefore, the pharmacotherapy for treating of obstructive pulmonary diseases including asthma is mainly based on inhaled bronchodilators associated with anti-inflammatory drugs such as corticosteroids. However, these drugs will cause some side effects and show limited therapeutic effects. So, there is an increasing demand for finding new treatment strategies in asthma therapy. A significant percentage of asthmatic patients use complementary and alternative medicine treatments, and herbal remedies have been introduced as one of the main choices for these patients. The results of a systematic review and meta-analysis study confirm that the supplemental use of herbal medicine in asthma patients markedly improved lung function compared to classic treatment without any significant side effects (Derakhshan et al., 2023).

Saffron (*Crocus sativus *L*., C. sativus*), a widely utilized Persian medicinal herbs, has shown promising effects in treating various diseases such as asthma. Saffron showed various pharmacological activities such as antioxidant (Abedi et al., 2023), anti-inflammatory (Boskabady et al., 2012), and immunomodulatory effects (Boskabady and Farkhondeh, 2016). The effects of saffron extract on the air way responsiveness in ovalbumin-sensitized animal were shown in previous studies (Byrami et al., 2013; Boskabady and Aslani, 2006). Based on the results of an experimental research, the addition of saffron extract to salbutamol can be considered an “add-on therapy” for asthmatics (Nair and Prabhavalkar, 2021). The therapeutic effects of saffron in asthmatic patients have been investigated in several clinical studies (Zilaee et al., 2019).

In comparison to other parts of saffron, the petal has fewer applications in food, cosmetics and the treatment of diseases. It has been shown that compounds and elements in saffron petal such as flavonoids and anthocyanins have biological effects and potential capacity in the treatment of diseases, and this can draw attention to saffron petal as a supplementary compound (Kazemi et al., 2023; Moshfegh et al., 2022). In addition, the petal is known as a source of active ingredients such as kaempferol and crocin, which have therapeutic properties.

Today, various studies are conducted with the aim of using medicinal plants in the treatment of obstructive pulmonary diseases. Hence, the current research was conducted to assess the relaxant property of *C. sativus* petal extract on rats tracheal smooth muscle (TSM) and its possible mechanisms.

## Materials and Methods

### Animals group

Fifty-four male Wistar rats (200±20 g) were kept at 22±2°Cand 50 to 60% humidity 12-hr with light and dark cycles in the Animal Breeding Center of Mashhad University of Medical Sciences, Mashhad, Iran. The experiments were approved by the Ethics Committee of Mashhad University of Medical Sciences (IR.MUMS.MEDICAL.REC.1398). Animals were randomly allocated in 8 groups as shown in Table 1.

### Preparation of tissues and smooth muscle relaxant testing

After anesthetizing rats by intraperitoneal (i.p.) administration of 50 ml/kg ketamine, they were sacrificed and their chest was opened. According to a previous study, bronchial rings were prepared and the relaxant effect of *C. sativus* petals extract on TSM was investigated (Memarzia et al., 2019). 

**Table 1:** Different studied groups including non-incubated tracheal smooth muscles (TSM) and incubated tissues. The relaxant effect of theophylline was examined only on two non-incubated groups.

**Group** **Abbreviation**	**n**	**Contractile agents**	**Condition**	**Incubating agent**	**Mechanisms**
NIT-Meta	6	Methacholine (10 μM)	Non-incubated tissues		
ITSM-Dilt	7		Incubated tissues	Diltiazem (5 μM)	Calcium channel blocking
ITSM-Glib	7			Glibenclamide (1 μM)	Potassium channel opening
ITSM-Prop	7			Propranolol (1 μM)	Β_2_-adrenoceptor blocking
NIT-KCl	6	KCl (60 mM)	Non-incubated tissues		
ITSM-Atro	7		Incubated tissues	Atropine (1 μM)	Muscarinic receptor inhibition
ITSM-Indo	7			Indomethacin (1 μM)	Cyclooxygenase inhibition
ITSM-Chlo	7			Chlorpheniramine (1 μM)	Histamine (H1) receptor inhibition

### Crocus sativus petal extract preparation

To prepare aqueous-alcoholic extract of *C. sativus*, saffron petals (50 g) were dried at room temperature away from sunlight and grounded. The powder obtained from saffron petal was dissolved with 50 ml of 50% ethanol (25 ml of distilled water and 25 ml of ethanol), shaken of 48 hr and the aqueous-alcoholic extract was obtained. Evaporation of the extract using a rotary evaporator at a temperature of 40°C was done to concentrate it (Memarzia et al., 2019).

### Evaluating the relaxant effect

According to the previous protocol, TSM contractions were induced by methacholine (10 M) or KCl (60 mM) for 5 min. Then, cumulative concentrations of *C. sativus *petal extract (0.1, 0.2, 0.4 and 0.8 mg/ml), theophylline (0.2, 0.4, 0.6 and 0.8 mM) or 1 ml normal saline (NS) were added to organ bath every 5 min and the relaxation response of each concentration was evaluated. Moreover, the concentration-response curve in each experiment was plotted and 50% of the maximum relaxation effect (EC_50_) was calculated. In the incubated groups, TSM was incubated 10 min before and following addition of various concentrations of *C. sativus* petal extract (Wang et al., 2017).

### Statistical analysis

Statistical analysis was done using one-way analysis of variance (ANOVA) followed by Tukey’s multiple comparison test by InStat (GraphPad Software, Inc., La Jolla, USA). Mean±SEM of the data are given and p values less than 0.05 were considered statistical significance.

## Results

### The relaxant effect of C. sativus petal extract on TSM contracted by methacholine

**Figure 1:**
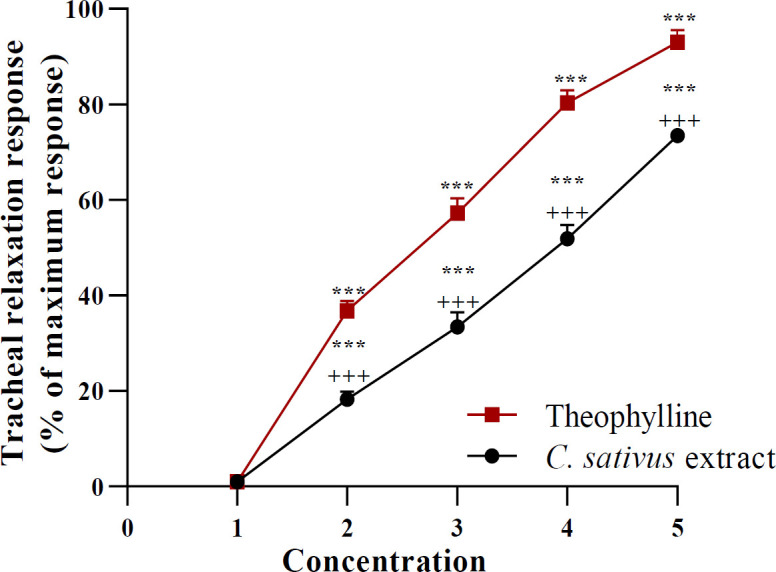
Concentration-response relaxant effects (mean±SEM) of 0.1, 0.2, 0.4 and 0.8 mg/ml C. sativus petal extract as well as 0.2, 0.4, 0.6 and 0.8 mM theophylline in tracheal smooth muscle (TSM) contracted by 10 µM methacholine. Four concentrations of the two agents are shown as 2, 3, 4 and 5 in X-axis, ***p<0.001 compared to the effect of saline (1 in X-axis). +++p<0.001 compared to the effect of theophylline. ANOVA with Tukey Kramer post-test was used for statistical comparison (All, n=6).

All concentrations of the extract produced relaxant effects in NIT-Meta group concentration-dependently (for all, p<0.001, Figure 1).

Various concentrations of the extract showed remarkably less relaxant effects in the NIT-Meta group compared to those of theophylline (for all, p<0.001, Figure 1).

**Figure 2:**
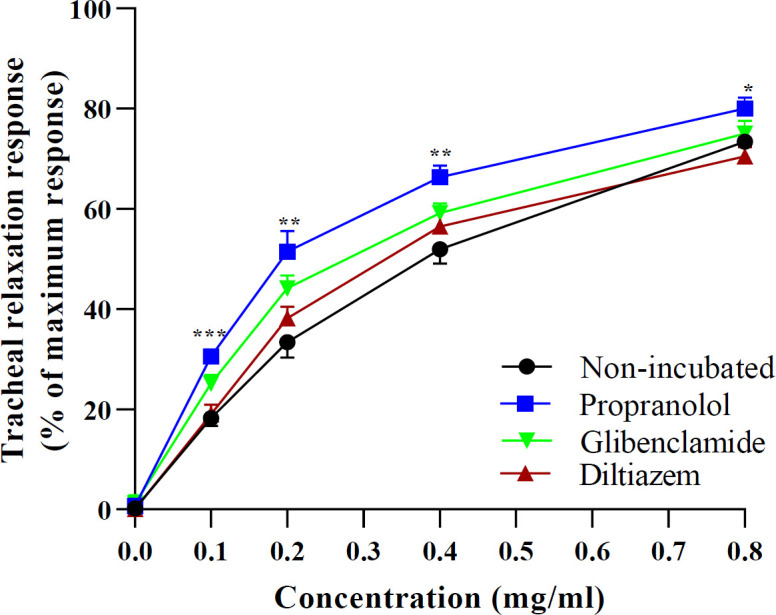
Concentration-response relaxant effects (mean±SEM) of 0.1, 0.2, 0.4 and 0.8 mg/ml extract of C. sativus petal in non-incubated TSM contracted by 10 µM methacholine (n=6), and TSM incubated with diltiazem, propranolol and glibenclamide (n=7). *p<0.05, **p<0.01 and ***p<0.001 compared to the effect of non-incubated TSM. ANOVA with Tukey Kramer post-test was used for statistical comparison**.**

**Figure 3:**
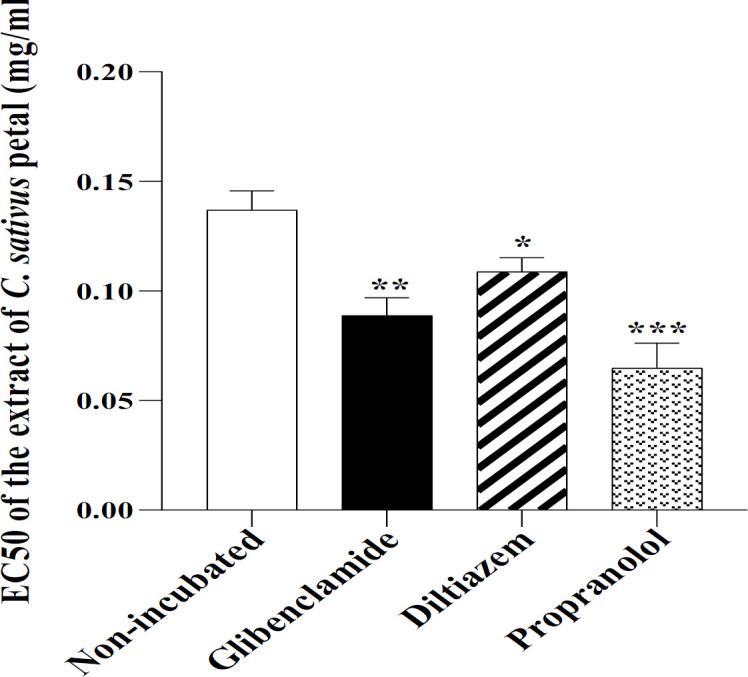
The level of EC50 of the extract of C. sativus petal in non-incubated TSM contracted by 10 µM methacholine, and TSM incubated with glibenclamide, diltiazem and propranolol. *p<0.05, **p<0.01 and ***p<0.001 compared to the non-incubated TSM. ANOVA with Tukey Kramer post-test was used for statistical comparison.

In the ITSM-prop group, all concentrations of the extract, showed greater relaxant effects compared with NIT-Meta group (p<0.05 to p<0.001, Figure 2). EC_50_ levels of extract in ITSM-Glib, ITSM-Prop and ITSM-Dilt groups were lower compared to the NIT-Meta group (p<0.05 to p<0.001, Figure 3).

### The relaxant effect of C. sativus extract on TSM contraction induced by KCl

**Figure 4:**
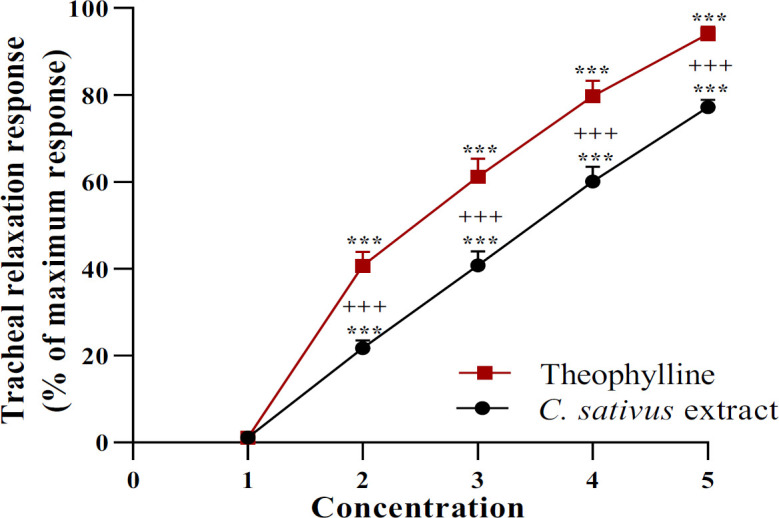
Concentration-response relaxant effects (mean±SEM) of 0.1, 0.2, 0.4 and 0.8 mg/ml C. sativus petal extract as well as 0.2, 0.4, 0.6 and 0.8 mM theophylline in tracheal smooth muscle (TSM) contracted by 60 µM KCl. Four concentrations of the two agents are presented as 2, 3, 4 and 5 in the X-axis. ***p<0.001 compared to the effect of saline (1 in X-axis). +++p<0.001 compared to the effect of theophylline. ANOVA with Tukey Kramer post-test was used for statistical comparison (All, n=6).

The relaxant effects of *C. sativus* petal extract in the NIT-KCl (60 µM) group were concentration-dependent and significant (for all concentration of the extract, p<0.001, Figure 4). In the NIT-KCl group, the relaxant effects of the all concentrations of the *C. sativus* petal extract were remarkably lower compared to those of theophylline (for all concentrations, p<0.001, Figure 4).

**Figure 5:**
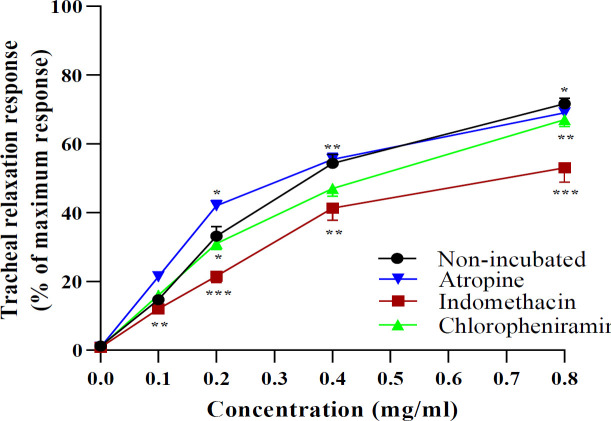
Concentration-response relaxant effects (mean±SEM) of 0.1, 0.2, 0.4 and 0.8 mg/ml extract of C. sativus petal in TSM contracted by 60 µM KCl (n=6) in non-incubated TSM, and TSM incubated with indomethacin, atropine and chlorpheniramine (n=7). *p<0.05, **p<0.01 and ***p<0.001 compared to the effect of non-incubated. ANOVA with Tukey Kramer post-test was used for statistical comparison.

**Figure 6:**
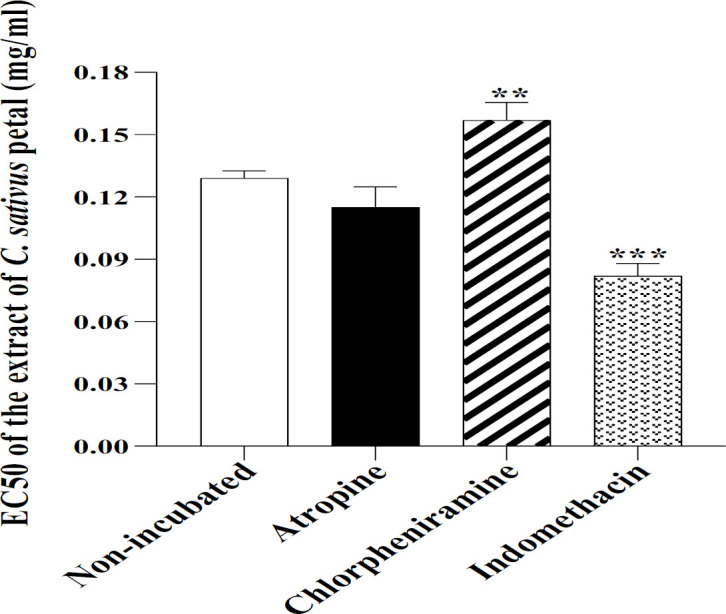
The level of EC50 of the extract of C. sativus petal in non-incubated TSM contracted by 60 µM KCl, and TSM incubated with atropine, indomethacin and chlorpheniramine. **p<0.01 and ***p<0.001 compared to the effect of non-incubated tissues. ANOVA with Tukey Kramer post-test was used for statistical comparison.

In the ITSM-indo group, the relaxant effects of all concentrations and in the ITSM-chlo group the effect of highest concentrations of the extract were less than NIT-KCl (p<0.01 to p<0.001, Figure 5). The ITSM-chlo group showed greater level of EC_50_ but the value obtained in ITSM-Indo was less than the NIT-KCl group (p<0.01 for chlorpheniramine and p<0.001 for indomethacin, Figure 6).

## Discussion

As far as we are aware, this work is the first study to report the significant relaxant effect of *C.* *sativus* petal on TSM contracted by methacholine or KCl. These results indicate that *C. sativus* petal could be introduced as a herbal-based product with bronchodilatory properties for the treatment of obstructive respiratory disease.

The study's findings demonstrated a significant concentration-dependent relaxant effect of *C. sativus* petal extract on non-incubated TSM that was contracted by contractile agents. The outcomes suggested that the extract had a remarkable relaxant effect which was lower than the effect of theophylline.

For assessing the effect of the extract on histamine, ß2-adrenergic, and muscarinic receptors, and potassium and, calcium channel as well as arachidonic acid metabolism, the relaxant effect of the extract was investigated on TSM incubated with chlorpheniramine, propranolol, atropine, glibenclamide, indomethacin, and diltiazem, respectively.

Among the mentioned groups, only in the presence of chlorpheniramine and indomethacin, the relaxant effect of the extract was significantly reduced, therefore, the inhibitory effect of the extract on histamine receptors and cyclooxygenase (COX) pathway are proposed as the possible mechanisms of the relaxant action of the extract. The lower EC_50 _values of the groups incubated with indomethacin and chlorpheniramine also support the inhibitory effect of the extract on histamine receptors and COX pathway. In asthmatic airways, metabolites resulting from the activity of COX enzymes such as prostaglandin D2 (PGD2), prostaglandin F2alpha (PGF2α), and thromboxane A2 can act as potent bronchoconstrictors via thromboxane receptors (Powell, 2021). In line with our finding, Peng et al. reported the inhibitory effect of the saffron petals on COX enzyme in an experimental model of colitis (Peng et al., 2023). Also, the modulating effect of the extract on the COX activity is in line with its anti-inflammatory properties which have been confirmed in previous studies (Kianmehr and Khazdair, 2020).

As a chemical mediator, histamine plays an essential role in asthma pathophysiology through regulation of the smooth muscle contraction in allergic reaction conditions. The finding of this study is consistent with our previous works on the inhibitory effect of saffron (Boskabady et al., 2010) and safranal (Boskabady et al., 2011) on histamine (H_1_) receptors in ASM. In our previous studies, we investigated other mechanisms involved in the relaxant effects of saffron on the respiratory airway, such as beta adrenergic stimulation (Nemati et al., 2008) and muscarinic receptors inhibition (Neamati and Boskabady, 2010), but these mechanisms did not play a role in the relaxant effects of the saffron petal extract.

Also, the relaxant effect of crocin on ASM involves mechanisms such as muscarinic receptor blocking, potassium channels opening and stimulation of ß2-adrenoreceptors ( Saadat et al., 2019). The lower EC_50 _values of the groups incubated with propranolol may also suggest the effect of the extract on stimulating ß2 -adrenergic receptors of TSM as a mechanism of the relaxant effect of saffron petal extract.

In addition, the relaxant action of saffron petal and its active ingredients on the smooth muscles of other tissues has also been investigated in another research. Fatehi et al. reported the relaxant effect of *C. sativus* petals extract on isolated rat vas deferens and guinea-pig ileum contracted with electrical field stimulation (Fatehi et al., 2003). In rat uterine smooth muscle, kaempferol showed a relaxant effect through cAMP that produces transcriptional and polyamines (Revuelta et al., 2000). According to Xu et al. study, the relaxant effect of kaempferol on porcine coronary artery smooth muscles is shown through activation of large-conductance Ca (2+)-inhibitory and K (+) channels opening (Xu et al., 2015). Kaempferol also relaxes rats pulmonary artery smooth muscle, which is mediated through inhibition of l-type calcium channels (Mahobiya et al., 2018).

In an experimental model of pulmonary arterial hypertension, crocin administration suppressed pulmonary arterial remodeling and reduced and improved mean pulmonary artery pressure (Sheng et al., 2022). In another study, inhibition of the release of intracellular Ca2+ stores in the endoplasmic reticulum was reported as a mechanism of the crocin relaxant effect on bovine aortic (He et al., 2004) and corpus cavernosum smooth muscles (Williams et al., 2005).

In this *ex-vivo* study, the relaxant effect of petal extract was examined in the presence of two contractile agents. Logically, *in vivo* studies are necessary to evaluate these effects in animal models of obstructive respiratory diseases such as asthma. In addition, we suggest that other mechanisms involved in the relaxant effects of petal extracts, emphasizing the biological properties of its active ingredient, should be considered in future studies. The findings of the present experimental research demonstrated the relatively potent relaxant effect of saffron petals extract on TSM although its effect was lower than theophylline at the concentration used. Inhibitory effects on histamine receptors and COX enzyme are suggested as mechanisms involved in the bronchodilatory effect of *C. sativus* petal.
